# Challenges for strengthening the health workforce in the Lao People’s Democratic Republic: perspectives from key stakeholders

**DOI:** 10.1186/s12960-016-0167-y

**Published:** 2016-11-29

**Authors:** Yi Qian, Fei Yan, Wei Wang, Shayna Clancy, Kongsap Akkhavong, Manithong Vonglokham, Somphou Outhensackda, Truls Østbye

**Affiliations:** 1 Department of Social Medicine, School of Public Health, Fudan University, 138 Yi Xue Yuan Road, Shanghai, 200032 People’s Republic of China; 2 Fudan Global Health Institute, Fudan University, Shanghai, 200032 People’s Republic of China; 3 Key Laboratory of Health Technology Assessment (Ministry of Health), Shanghai, 200032 People’s Republic of China; 4 Department of Community and Family Medicine, Duke University, Durham, NC United States of America; 5 National Institute of Public Health, Vientiane Capital, Lao People’s Democratic Republic; 6 Center for Aging Research and Education, Duke-NUS Medical School, Singapore, Singapore; 7 Duke Global Health Institute, Duke University, Durham, NC United States of America

**Keywords:** Health workforce, Lao People’s Democratic Republic, Qualitative research

## Abstract

**Background:**

The Lao People’s Democratic Republic is facing a critical shortage and maldistribution of health workers. Strengthening of the health workforce has been adopted as one of the five priorities of the National Health Sector Strategy (2013–2025). This study aims to identify, explore, and better understand the key challenges for strengthening the Laotian health workforce.

**Methods:**

This study applied exploratory and descriptive qualitative methods and adapted a working life-span framework. Twenty-three key stakeholders with particular insights into the current situation of the health workforce were purposively recruited for in-depth interviews. Important policy documents were also collected from key informants during the interviews. Thematic analysis was employed for the textual data using MAXQDA 10.

**Results:**

The overarching problem is that there is a perceived severe shortage of skilled health workers (doctors, nurses, and midwives) and lab technicians, especially in primary health facilities and rural areas. Key informants also identified five problems: insufficient production of health workers both in quantity and quality, a limited national budget to recruit enough health staff and provide sufficient and equitable salaries and incentives, limited management capacity, poor recruitment for work in rural areas, and lack of well-designed continuing education programs for professional development. These problems are interrelated, both in how the issues arise and in the effect they have on one another.

**Conclusions:**

To improve the distribution of health workers in rural areas, strategies for increasing production and strengthening retention should be well integrated for better effectiveness. It is also essential to take the Laotian-specific context into consideration during intervention development and implementation. Furthermore, the government should acknowledge the inadequate health management capacity and invest to improve human resource management capacity at all levels. Finally, assessment of interventions for health workforce strengthening should be developed as early as possible to learn from the experiences and lessons in the Lao People’s Democratic Republic.

**Electronic supplementary material:**

The online version of this article (doi:10.1186/s12960-016-0167-y) contains supplementary material, which is available to authorized users.

## Background

### Human resources for health: a global picture

Optimal health cannot be achieved without a health workforce, since health personnel represent the human linkage connecting health knowledge to health action [1–3]. The health workforce is at the heart of all health systems and plays a key role in improving health services and achieving health goals [1–3]. However, the global health workforce is in a severe crisis. It has been estimated that the global deficit of skilled health workers (doctors, nurses, and midwives) has increased from 2.4 million in 2006 to 7.2 million in 2012 and is expected to reach to 12.9 million by 2035 [1, 3]. Moreover, uneven distribution across the globe and within countries is another big challenge [1, 3]. The countries who suffer greater burden of disease and require more sufficient health staff usually have more severe shortage of health workers. Within countries, the inequitable distribution of health workers is also evident between urban and rural areas. Furthermore, there is evidence indicating that the global health workforce is aging [3]. It is therefore important to rethink the models of education, deployment, and remuneration of the health workforce and start a new global agenda relating to human resources for health development to reinforce the health workforce based on better evidence and practices.

### The health workforce in the Lao People’s Democratic Republic

The Lao People’s Democratic Republic is one of 57 countries having a critical shortage of health workers. The country only had 14 189 health workers in 2012 [1, 4, 5]. Of those health workers, 59% were female and 16% were from minority groups [4]. Between 2005 and 2012, the average number of skilled health workers per 1000 population was 0.2 physicians and 0.8 nursing and midwifery personnel [5]. This number was far below the minimum threshold of 2.28 skilled health workers per 1000 population recommended by WHO [1]. Medical doctors, nurses, and midwives with middle and high levels of education only accounted for less than 30% of total health workers [4]. There is therefore a critical shortage of well-trained health workers, especially the qualified medical staff.

Health workers in the Lao People’s Democratic Republic are distributed unevenly among different provinces. Between 2009 and 2010, the capital had the highest number of health workers per 1000 population, 4.2 for all types of health workers, twice the number of health personnel per 1000 population at the national level during the same period [4]. Moreover, maldistribution of health workers also exists among different health facility types (central hospitals, regional hospitals, provincial hospitals, district hospitals, and village health centers), with most being employed in district hospitals and provincial hospitals [6]. Few health workers take positions in village health centers, which are mostly located in remote, mountainous, and hard-to-reach areas [4].

Additionally, reports indicate that the overall capacity of health workers in the Lao People’s Democratic Republic has limited competence due to poor quality of medical training and limited incentives including low salaries and lack of opportunities for professional development, which also affect the performance of health staff [5, 6].

These human resource constraints in the Lao People’s Democratic Republic are similar to those in many other developing and developed countries. Australia has more doctors and registered nurses working in metropolitan areas than in remote areas; thus, the Australian government launched the “More Doctors, Better Services” strategy in 2000 to address the shortage of health personnel in rural regions [7]. In China, health workers are distributed unevenly, not only between rural and urban locations, but also among the regions [8]. Thailand is also experiencing internal migration of skilled health professionals from rural to urban areas and from the public to the private sector, posing a new challenge to healthcare [9].

### The health system in the Lao People’s Democratic Republic

The health system in the Lao People’s Democratic Republic operates at three administrative levels: central (Ministry of Health, MOH); provincial (provincial health offices, PHOs); and district level (district health offices, DHOs) [4, 6, 10]. The Laotian health system has been decentralized, and some planning and budgeting responsibility have been devolved to the provincial and district levels, but there is evidence that health management and leadership capacity at the provincial and district levels is limited [4–6]. The health-related expenditures from the government are also modest, and the health system significantly relies on external funding from donors [6, 11]. The government only allocated 2.6% of total expenditures to the health sector in 2012 [12]. Healthcare in the Lao People’s Democratic Republic is predominately delivered by public healthcare providers, at four levels of organization: hospitals at the central level managed directly by the MOH, hospitals at the provincial level managed by the PHOs, hospitals at the district level managed by the DHOs, and providers at the community level (health centers and village drug kits) also managed by the DHOs [4, 10, 13]. There are also a large number of private pharmacies and clinics, but no private hospitals [4]. The uneven distribution of the health workforce geographically and by facility type has left many primary healthcare facilities understaffed and unable to provide basic services [6]. The structure of the healthcare facilities is shown in Additional file 1.

The Lao People’s Democratic Republic is now under the 7th National Socio-Economic Plan and utilized the 7th Five-Year National Health Sector Development Plan as a roadmap to achieve the health-related Millennium Development Goals (MDGs) and improve the life of all Laotians [14]. Under this framework, the National Health Sector Strategy (2013–2025) was approved in 2012 and would be implemented in three phases, aiming to achieve the health-related MDGs by 2015 and universal health coverage by 2025. Health workforce strengthening is one of the five priorities for reform [10]. Hence, developing a better understanding of the current health workforce situation in the Lao People’s Democratic Republic is necessary for achieving the human resources for health target. Previous research and reports have mostly included quantitative information on the health workforce in the Lao People’s Democratic Republic and have not included a comprehensive and integrated picture of the health workforce in the Lao People’s Democratic Republic. This study aims to identify, explore, and better understand the key challenges for strengthening the Laotian health workforce. We also provide recommendations on further health workforce development in the context of health system reform in the Lao People’s Democratic Republic.

## Methods

### Study design and framework

This study was designed to utilize exploratory and descriptive qualitative methods as it was the best option for achieving the scope of this study [15], mainly through the in-depth interviews (IDIs) with key informants. These interviews aimed to explore and gain better understanding of the problems and challenges faced by the national healthcare workforce in the Lao People’s Democratic Republic.

**Fig. 1:**
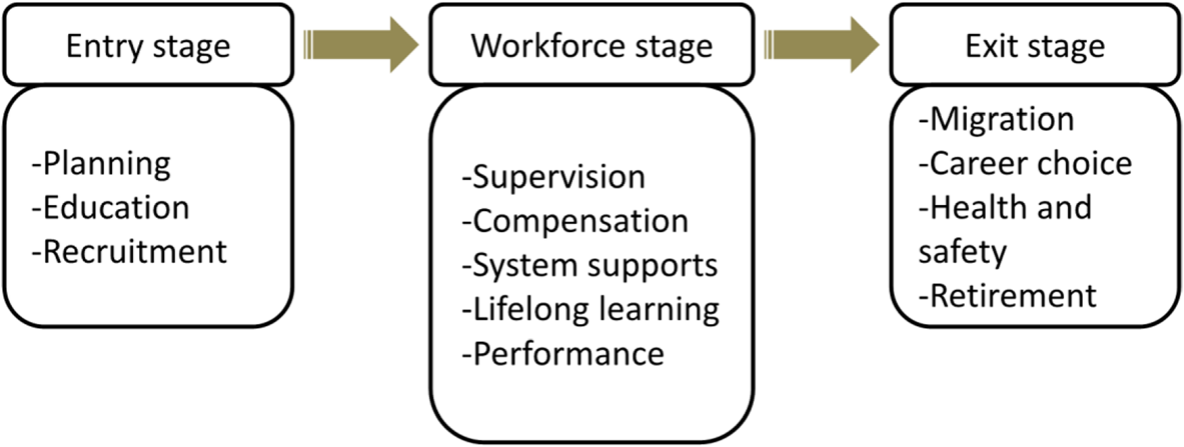
The working life-span of entry-workforce-exit framework. Note: adapted from [1]

We adapted the “working lifespan of entry-workforce-exit” framework from the World Health Report 2006, which focuses on “three stages”: (1) when people enter the workforce (entry stage), (2) when they are part of the workforce (workforce stage), and (3) when they exit from the workforce (exit stage) [1] (see Fig. 1). In the entry stage, there are three central aspects of interest: planning, education, and recruitment. The workforce stage assesses the following aspects: supervision, compensation, systems supports, lifelong learning, and performance. Finally, migration, career choice, health and safety, and retirement are the four key aspects in the exit stage; these are also the influencing factors of workforce attrition.

### Sampling of key informants

Key stakeholders with particular insights into the current health workforce situation were recruited through a purposive sampling strategy [15]. The sampling strategy had taken into account the accessibility, convenience, and availability of the key informants.

Study sites in both urban and rural areas and with different levels of economic conditions were selected. Two provinces were first selected: the Vientiane capital and the Vientiane province. The Vientiane capital is an urban region, and the Vientiane province is a rural region. The former is more economically developed than the latter. The Xaythany district in the Vientiane capital and the Hinheub district and Vangvieng district in the Vientiane province were then selected for recruitment of key informants.

**Fig. 2:**
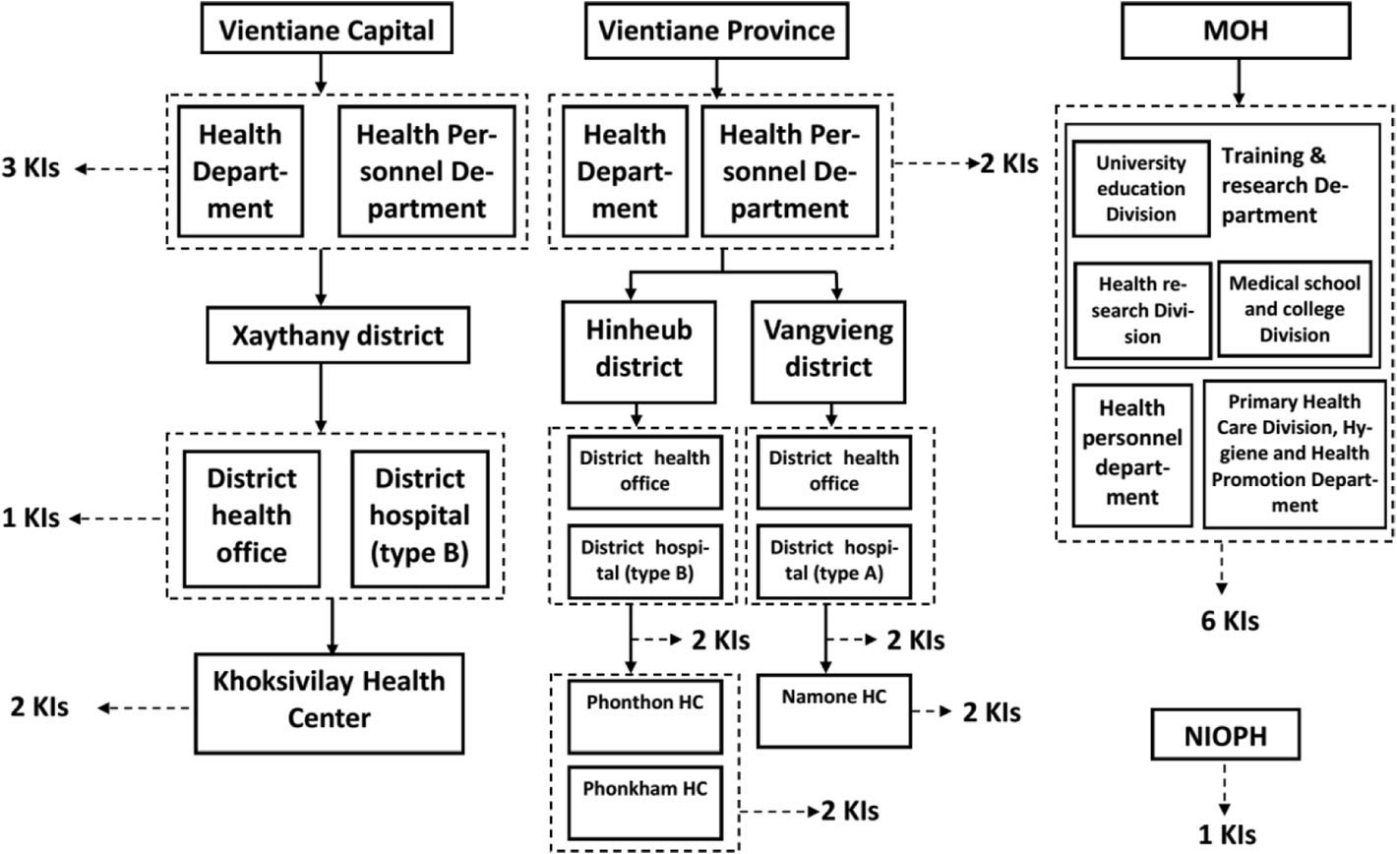
Selection of key informants. *HC* health center, *KIs* key informants

Three categories of key informants were recruited for this study: policy makers, administrative staff, and medical staff. The policy makers in positions for national health workforce planning and management were selected from the MOH. The administrative staff involved in human resources for health management was enlisted from three levels: (1) administrative staff from the central level (MOH and National Institute of Public Health, NIOPH), (2) administrative staff from the provincial or district level, (3) administrative staff from a variety of health facilities (type A and type B district hospital and health centers). Medical staff with different experiences in provision of healthcare were also recruited. The sampling process is showed in more detail in Fig. 2.

### Data collection

The fieldwork was conducted over 3 weeks in the Lao People’s Democratic Republic. The first 1-week period was used by the Lao People’s Democratic Republic, Chinese, and US research team members to prepare for fieldwork and conduct a pilot interview. The last 2 weeks (November, 2014) were utilized for key informant interviews.

Four topic guides for different types of key informants (policy makers and administrative staff from the central level, administrative staff from the provincial or district level, administrative staff from health facilities, and medical staff) were used to facilitate the interviews. The topic guides were designed to cover the key aspects included in the working life-span framework. The main points for probing for the interviews are presented in Additional file 2. Furthermore, the important policy documents over and beyond those that the researchers had already identified from the literature review were also collected from key informants during interviews.

IDIs were conducted by the primary researcher in English and Lao with a research assistant fluent in both English and Lao and with experience in public health research. An active interpreter model was applied during the interviews: the researcher facilitated the IDIs in English, and the research assistant translated and summarized the key responses from participants to the primary researcher to allow her to ask additional questions [16]. The key informant interviews were terminated when there was no new information forthcoming, most of which lasted 1.5 to 2 h. The final sample size was reached by saturation of information [17, 18]. Most of the interviews were recorded using a digital voice recorder, and field notes were taken during the interviews (two interviews were done without recording but with detailed field notes).

There was a total of 23 key informants interviewed. A broad range of policy makers and administrative staff were interviewed. The different key informants are presented in Table 1. The Health Personnel Development Strategy by 2020 and Handbook of Health Professions and Educational Programs were key policy documents gathered.

**Table 1:** Key informants interviewed

	Policy makers	Administrative staff	Medical staff
Central level	7	–	–
Provincial level	–	5	–
District level	–	3	–
Hospital	–	2	–
Health center	–	4	2
Subtotal	7	14	2

### Data analysis

A thematic analysis was employed for the textual data collected using MAXQDA 10 [15]. The recordings were transcribed verbatim in English and then double-checked by a research assistant for accuracy of transcriptions. The themes were developed mainly based on the guiding framework and also generated from the topic guides and the textual data of the IDIs. Information from policy documents and IDIs were all segmented by themes. The analysis process was inductive: themes were identified, coded, classified, and recoded, with the data becoming themes. The themes and codes were revised and refined continuously and integrated providing new insights.

### Data quality assurance

The research team had attended the training on qualitative research and had experiences of qualitative fieldwork. Triangulation was applied to explore the issues from different sources: policy makers, administrative staff, and medical staff. Moreover, key informants had various backgrounds, characteristics, and experiences reflecting a broad range of perspectives. Furthermore, open-ended question guides were used to capture the range and depth of the perspectives. Interviews were conducted until no new information was elicited. During the interviews, the replies were validated with the respondents to ensure that the main issues have been captured. The methods and process of data collection and analysis were written up, helping to increase the transparency of this study.

### Ethical considerations

Ethical approval was obtained from the Ethics Committee of the School of Public Health of Fudan University (Reference number: IRB#2014-09-0532). The consent forms in Lao with explicit explanation of the study, confidentiality, privacy, and anonymity were provided to all participants before the interviews. Participation in the study was voluntary and confidentiality was assured. Participants agreed to take part in the study and signed the informed consent forms. Additionally, permission for recording was also obtained from all participants before the IDIs. All paper data and digital data were appropriately stored under lock or were password protected.

## Results

### Current stock and demand for health workers: severe shortage of skilled health personnel

The key informants from the Health Personnel Department of the MOH stated that there were two main types of health workers in the Lao People’s Democratic Republic: civil servant health workers and contract health workers. The civil servant health worker holds an official position in the public health sector and receives a stable salary, whereas the contract health staff has no official position and no salary.

Most key informants reported the biggest problem to be the severe shortage in health workers, especially in rural and remote areas. The greatest deficits were for skilled health personnel and laboratory technicians. Furthermore, most highly qualified health personnel worked at central- and provincial-level health facilities, not at the primary health level. Key informants attributed the shortage to the insufficient budget at the national levels for civil servant positions and poor attraction to work in health centers and rural areas.

…one thing is they want to put the bachelors, bachelor graduates in health centers, but there is no bachelor, there are only medical assistants, so they have to put the medical assistants to work in the health centers, that is the problem. (IDI 1)

### Personal incentives: insufficient and inequitable

Most of the key informants reported that the incentives provided to health workers were insufficient and inequitable across different types of health staff. The salary level of civil servant health workers mainly depended on their “rank,” which was determined by the Ministry of Finance according to their education background and working experiences. This implied that civil servant health workers of the same rank would receive similar salaries no matter which type of facility they work in. Retired civil servant health workers receive around 60–70% of their original salary monthly.

**Fig. 3:**
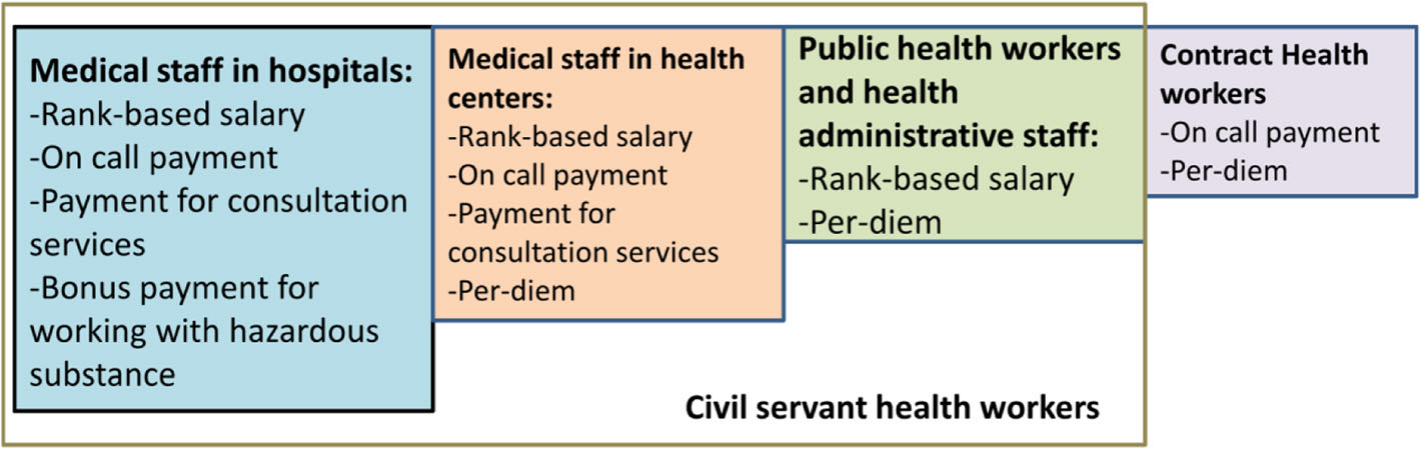
Summary of income structures for different types of health workers in the Lao People’s Democratic Republic

Besides rank-based salary, many key informants considered that the other financial incentives were not equitable across different types of civil servant health workers (see Fig. 3). Medical staff in hospitals could also gain financial incentives from other sources, including bonus payments for on-call hours, working with hazardous substances, and consultation services. The latter two types of bonus payments were contingent on hospital funds and were not stable and reliable sources of income. The health workers in health centers could only get bonus payments for on-call hours, consultation services, and per-diem payments for outreach services. One administrative staff reported that some health center staff could earn extra income by working part time in private pharmacies or clinics.

The public health workers and health administrative staff only got rank-based salary and per diem. The contract health workers only received payments for on-call hours and per diem work. Due to the inconsistency of work hours, most of them need to rely on their family to survive.

Because they are local people, living in that village, that means that the health center is located in that village, it is very near to his house, so even their salary is not high, but they are not in a farming situation, because their parents are there, when their salary is not sufficient, they can go to the parents, so the parents will give them (food…), so they will not die. (IDI 2)

In addition to financial incentives, the health workers who were evaluated as “outstanding” in performance appraisals were provided with upgraded rank, participation in medical or degree training, and enrollment in medical conferences as additional non-financial incentives.

Furthermore, their payments may be delayed by months and most participants considered this as a main concern. The payments would be delayed due to inadequate governmental management ability and complicated processes for fundraising and appropriations. The health workers would often rely on their families for living expenses during unpaid periods.

we (health center) get from the district, and the district gets from provincial health department, and the provincial health department gets from provincial financial department. The payment is delayed, we get only up to the October payment, so from November until now, we did not get paid. (*IDI 7 conducted in March, implying the staff didn’t receive payments for 4 months*)

### Performance of health workers: perceived conscientious and dedicated

Most key informants thought the health workers, especially those in health centers, were conscientious and dedicated to their work, even during the unpaid periods. Furthermore, community members were perceived to be satisfied with the health services and have developed good relationships with health workers.

And our staff are devoted to the villages, although we don’t get anything [salary and incentives], we still go for the outreach activities, and the villagers also like us, when we are working in the villages, even though we don’t get any support from the district health office, the villagers would provide food to us. (IDI 3)

The two most important factors related to performance were locality and staff assignment. Due to easier living conditions and support from family, local staff might perform better than non-local staff. The MOH may assign a health worker to a less desirable area, even if they are not qualified for that position out of necessity due to the shortage of skilled health professionals. In addition, several participants mentioned other factors that impact performance including salary level, health insurance, housing conditions, and personal reputation.

Locality, is most important factor, they are close to their family, they can help their family, they are living in the village where the health center is located, so they can go to the health center easily. (IDI 8)

### Performance appraisal: lack of standardized measurements and influences on health workers

According to the discourse of policy makers, performance appraisal was implemented at two levels: the institutional level and the health staff level. The performance appraisals of health facilities were conducted using standard checklists by external upper-level health administrative departments, mainly covering management, financial, and technical aspects. However, there was a lack of consistent and detailed knowledge on the evaluation checklists. The performance appraisal reports were also unavailable for us to review.

One problem reported by some policy makers was there was no standardized method of evaluating health staff; thus, health facilities were left to create their own evaluation systems covering different aspects. One district hospital followed the evaluation system created by an international organization and adopted several dimensions including clinical skills, interpersonal relationship both with colleagues and patients, attendance, compliance with professional regulations, and attitude. Some health centers used the feedback from the community, and some did not have any performance appraisal at all.

Most key informants indicated that performance appraisal produced limited influences on health workers. Although there is a chance that employees may move up in rank sooner than the standard 2 years, this is only for those deemed outstanding and it is uncommon. Rather than being terminated, poorly performing staff were required to reflect on their performance and were trained on how to adjust their actions in the future. Poor performance had few and only minor repercussions.

… normally every two years, you would be promoted one sub-rank, your sub-rank would be increased by one, but if your performance is outstanding, you have never done any mistakes, the hospital would propose to the provincial health department, and then to the MOH, to upgrade your sub-rank of one, so instead of waiting for two years, you do it only one year, you get it only one year. (IDI 6)

…so you ask about the impacts of the evaluation: there is no penalty the staff, we don’t fine the staff, once you are doing wrong, we would call that staff to tell what you have been doing, that is the first stage, and the second stage is that, if the staff member need to write down what he had done wrong, then he must promise that he would not do it again, and he would change his behavior, his performance; and if it is the third stage, in two years, we would upgrade the rank, he would not be upgraded, but so far, there is no health work at stage 2 or 3, only stage 1. And sometimes, the staff moves away to another department, but it is very rare. (IDI 8)

### Education and continuing education: completely established but disconnected from health workforce demand

The “Handbook of health professions and educational programs” indicates that the Lao People’s Democratic Republic has developed a complicated health profession educational system, covering detailed core competencies, curriculum structure, and career options for each category [19]. The education system was designed to train four main classifications of staff including medical and dental staff, nursing and midwifery, and paramedical staff, as well as managerial and support staff [19]. The former three categories had five levels of education: low level, middle level, high level, bachelor level, and postgraduate level [19]. The duration of programs varied among disciplines from 2 to 6 years [19]. The policy makers reflected that the annual number of graduates in all categories was around 2000, from 10 health education institutes (1 medical university, 5 health professional colleges, and 4 health schools).

One problem mentioned by several policy makers was that the training of low-level health workers has been discontinued since the government planned to improve the education level of the health workforce by no longer producing low-level health workers. Meanwhile, the existing low-level health staff were required to upgrade education levels through continuing education courses. However, some current low-level health staff had difficulties in upgrading because they were elderly.

Some of the education programs were provided to current health workers as long-term continuing education programs but in shorter duration than the direct entry programs. The short-term continuing education programs were conducted at four levels: health facility, district, provincial, and central levels. The programs at the health facility level consisted of on-site training with varied frequencies among facilities. The district-, provincial-, and central-level programs were held in the form of centralized training covering different topics. Some international organizations also provided trainings or funding to support training programs. In addition, health staff would be sent to upper-level health facilities or other countries for long-term training, mostly depending on scholarship opportunities. Most of the participants held positive perceptions on these programs and desired more training to improve knowledge and skills.

In terms of continuing education, some key informants reflected that attending continuing education programs were not obligatory for health staffs to maintain their license. The service need of health facilities and the availability of funding to support the training are the key influencing factors. Participation in continuing education programs were occasionally offered to outstanding health staff as incentives.

The main concern of most interviewees on medical education was the disconnect between medical education and health workforce demand. The medical education programs were not based on the actual demand, and the students in health disciplines were trained in less relevant disciplines. Furthermore, health education facilities and teachers were lacking, and the students even did not have sufficient opportunities to conduct clinical practices. Therefore, the medical education programs were limited in quality and insufficient to train competent health workers.

…so you accept 30 persons without planning, you just train and train without goal, what are you going to use these people to do, you don’t train according to your need, what is why… (IDI 2)

### Recruitment and hiring: highly competitive for limited positions

Both the policy makers and administrative staff reported that there were two types of patterns to recruit health workers: top-down from the MOH and bottom-up from within the health facilities. The top-down recruitment included health staff who were sent from the MOH to health facilities. The procedure for top-down recruitment was to make an announcement, hold exams and interviews for candidates, rank and recruit the eligible candidates based on available civil servant positions, and assign positions. The candidates were required to pass an exam from the Ministry of Home Affairs and an exam from the MOH to work in the health sectors. Many participants said that locality was the key factor for the MOH to allocate health workers to specific health facilities. Although the MOH considered the preferences of candidates, they preferred to send the graduates back to the health facilities in their home regions. The assignment of positions was mostly decided by district health offices. Most of the top-down recruited health personnel had civil servant positions.

Bottom-up recruitment occurs when candidates who could not obtain a civil servant position through the MOH apply directly to health facilities for contract positions. Then the health facilities can apply for a civil servant position from the MOH for this staff, but this process could take several years while the staff work in a contract position. Most medical graduates would apply through the top-down system because they could directly receive the civil servant position with salary from the MOH rather than applying through the bottom-up pattern to get contract positions without salaries.

Some administrative staff reflected that only about half of the medical graduates could obtain civil servant employment through the MOH and the rest usually became contract health staff in health facilities because the current available civil servant positions are insufficient to absorb those already trained.

### Mobility of health workers (attraction, retention, and loss): low attractiveness but good retention in primary health facilities and rural areas

Most key informants mentioned that many health professionals preferred to work in health facilities of the central or provincial levels rather than the primary health facilities such as health centers or health facilities in rural areas. The low attractiveness of working in primary health facilities and rural areas was a big concern. The reasons for this included the following: no housing supplied to non-local health staff, poor living conditions, inconvenience in commuting because of the poor transportation system, and the lack of confidence to practice alone without supervision in rural areas. One participant shared that many new graduates felt challenged and unprepared to take positions in rural areas because they were supposed to deal with all medical conditions independently, including very complicated medical cases. The health workers who were willing to take the positions in rural areas were mainly because they were from the local, and they could gain the civil servant positions there.

… this is the experiences when we collected data in Vientiane province, they don’t believe in themselves to work in the rural area, because the new graduates, you need to “wear many hats” in the rural area, “I am a medical doctor, I am not a surgeon, I am not a pediatrician”, but when you are posted in a health center, when the patient comes, you can’t say no, I can’t do with that”, you have to be able to…even operate on the patient, that is why they are not confident… (IDI 11)

Most interviewees agreed that the general retention of health workers was good, including the health workers in rural areas. Health workers who had local families and had developed good relationships with patients and local communities were more likely to stay in their positions. Some health workers regarded the stability of career and the reputation in society as important for retention. Moving with family was the primary reason for health staff to change their jobs. One MOH staff recommended providing bonuses and supportive supervision in medical practices to rural health workers to better support them working in remote areas.

### Insufficient financial support for the health workforce and for health facilities

Most key informants stated that the main financial support for operation of health facilities came from the government, including costs of health personnel salaries, on-call payments, infrastructure, and basic medical equipment. The funding for outreach services in health centers came mainly from the district government. In addition, some international organizations or companies fund health facilities for outreach services or specific health promotion events. The funding for outreach services or health promotion events was allocated quarterly and required health centers to submit plans including their budget.

…before, we got the budget from two sources, one was the district health office, the other from the Lao Luxemburg Development Organization, this is in collaboration between Lao government and Luxemburg government, they provide the funds quarterly, depending on the activities of health centers, so when you have activities, you write down your plans, then you submit your plans to the Lao-Luxemburg Development Organization, when they check and approve, so they provide the funding support according to the activities. But currently we get from one source, the district health office. (IDI 3)

Most key informants considered the insufficient budget for the health workforce to be a problem. The government could not afford sufficient civil servant positions to hire the medical graduates, and the salary and incentives provided to current health workers were insufficient. Simultaneously, most directors of health facilities stated that the financial support from the government was also insufficient, so this became a key constraint for health facilities to provide health services with a limited budget. Health centers could use the profits from sale of medications or from charging patients for services to pay for utilities or incentives to health workers. However, the usage of profits more than 1 million kip (≈120 US dollars) per month required the approval from the district health office.

One administrative staff member reported that there was poor integration between external funding from international organizations or companies and the government, which led to the funding not being spent wisely. The external funding from international organizations or companies and government funding were spent on similar activities without coordination so they duplicated efforts, indicating the poor capacity of funding management.

…the World Bank provided the money for the per diem, for food, accommodation if they stayed overnight in the community for outreach work, and transportation means fuel to the outreach team, and the outreach team would ask them to integrate with the routine of the government, that was the district plan to go every 3 months, that means 4 times a year, in order to cover the immunization and the Vitamin A supplementation, and the MCH. But instead of doing so, the government didn’t put the money together to run outreach activity services and world bank went this time, so the government went again using the governmental money for the same, and they came back, the next week, they would go with other funding, you see… (IDI 7)

## Discussion

### The interrelationships among the problems

**Fig. 4:**
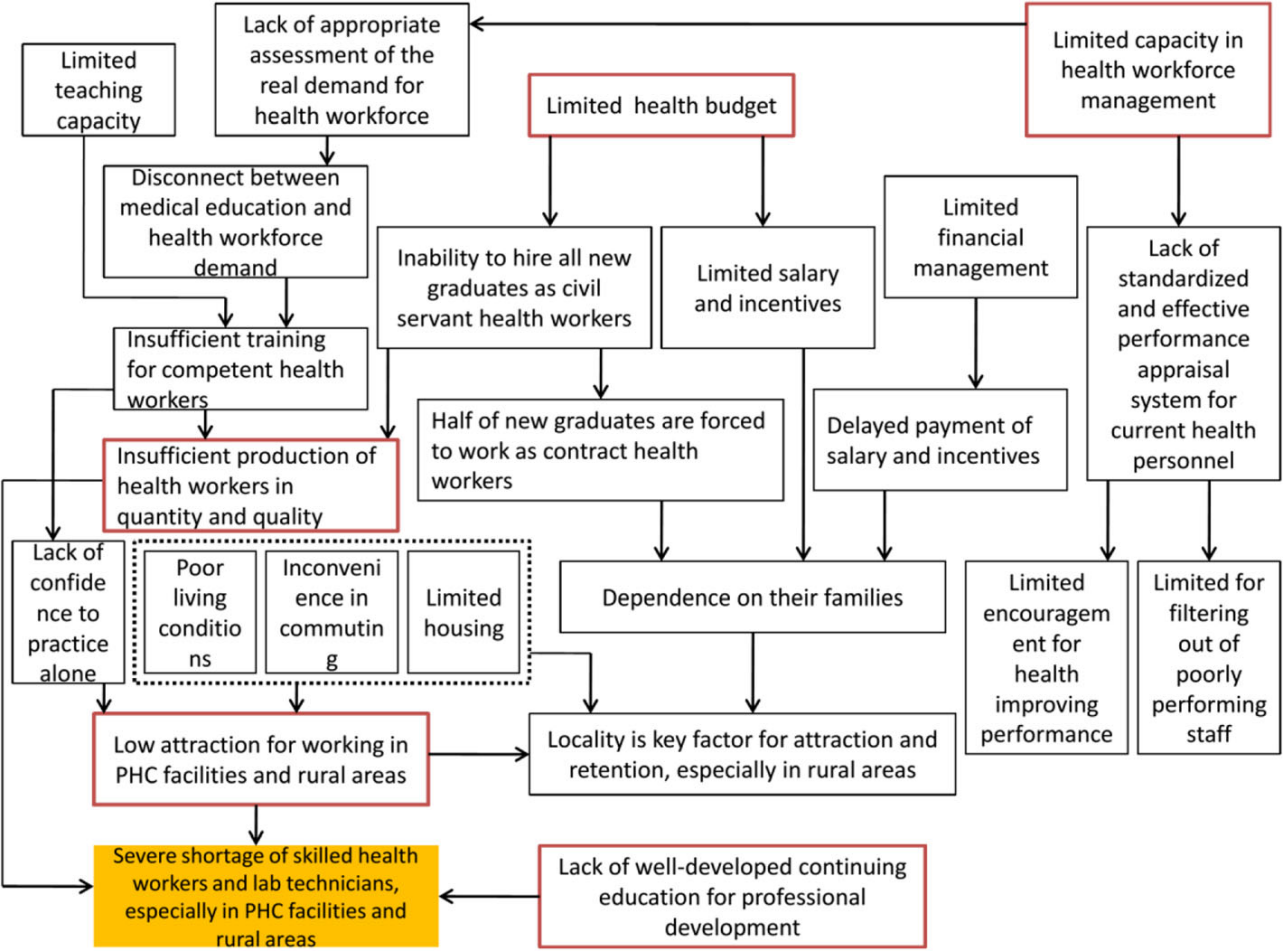
Interrelationships among key problems in the health workforce in the Lao People’s Democratic Republic. Note: skilled health workers: including doctors, nurses, and midwives; *PHC* primary healthcare

The key aspects explored in the IDIs provide a picture of the current status of the health workforce in the Lao People’s Democratic Republic and reflects the problems of the health workforce using *the working life-span framework*. The perceived overarching problem is that there is a severe deficit of skilled health workers (doctors, nurses, and midwives) and lab technicians, especially in primary health facilities and rural areas. This deficiency is related to five main problems discussed by the key informants. First, the production of health workers is insufficient both in quantity and quality. Second, the national budget is inadequate to recruit enough health workers and provide sufficient and equitable salaries and incentives to them and the payment is often delayed. Third, the health workforce management is weak, and this lack of capacity is a major barrier to support the health workforce. Fourth, while rural areas have good health workforce retention, especially among the health workers of local origin, these areas are not attractive to most outsiders. Last but not least, there is a lack of well-developed continuing education programs for professional development. These problems are interrelated, both in how the issues arise and in the effect they have on one another. (The five main problems are marked in red boxes in Fig. 4.)

There are two possible reasons for the *insufficient production of health staff both in quantity and quality. As a consequence of inadequate capacity in health workforce management, no appropriate assessment is adopted in health workforce planning* to reflect the real demand for health workers. Thus, the medical education programs are not based on the real demand, and most of them are inadequate to produce health workers with adequate knowledge and skills. This is especially true for those who are to practice in rural areas where health workers are required to deal with all medical conditions independently. Meanwhile, the available number of health education facilities and teachers is insufficient to train competent health workers, but this situation is disregarded during the recruitment of new students.

*The MOH has limited budget to recruit all new graduates as civil servant health workers and to supply sufficient salary and incentives. *As a result of this, almost half of new graduates are forced to work as contract health workers who receive low and unstable payment. Additionally, salaries and incentives are often delayed for months because of the inadequate capacity in financial management and complicated processes for fundraising and appropriations. The insufficient and delayed payment forces health personnel to depend on their families; therefore, locality becomes a significant factor in attraction and retention, especially in rural areas.

There are four potential reasons for *the low attraction of working in primary health facilities and rural areas*. There is no housing supplied to non-local health staff, poor living conditions, and inconvenient commuting because of the poor transportation system. Furthermore, the health workers may lack confidence to practice alone without supervision in rural areas.

*In addition, the current performance appraisal system does not* notably encourage health personnel to improve their performance and filter out poorly performing staff.

### Priorities to strengthen the health workforce

#### Tailored and integrated interventions to address uneven distribution of health workers

Uneven distribution between urban and rural regions should remain a policy priority for improving the health workforce in the Lao People’s Democratic Republic since the shortage of qualified health personnel in remote and rural areas can hinder a large proportion of the population access to health services and lead to disparities in health outcomes between those in rural and urban areas [20, 21]. Many countries have a similar maldistribution problem and emphasize the training of more rural health workers. However, experiences from other countries show that production of more medical staff is insufficient to improve the distribution of health personnel. In Nepal, new medical schools were opened to increase the supply of rural health workers, but this leads to overproduction and emigration of medical students [21]. China launched a rural doctors project to improve the staffing of rural health centers in 2010, providing candidates with a scholarship package including free tuition, room, and living expenses but requiring them to serve 6 years at their local health center. However, there was a high turnover rate among the graduates in this program, who were still attracted to work in upper-level health facilities in urban locations [22]. The Indonesian government implemented a compulsory services project to increase the health workers in rural areas, which found that female candidates were less likely to be prepared to serve in remote areas even if they were paid double the salary of health staff working in urban areas [23, 24]. These suggest that increased production should be combined with retention strategies.

The Lao People’s Democratic Republic has its own specific characteristics and determinants, which should be taken into consideration for HRH strategy development to ensure that the choice of interventions is tailored to the local context. As discussed earlier, it is not so attractive to work in rural regions but there is still a fairly good retention of health workers in these regions. Therefore, it may be practical to admit more students with rural backgrounds because personal origin is a key factor both in attraction and retention for these workers in rural regions. In addition to the factors on low attraction identified in this study, it has also been highlighted in previous reports that new graduates of health-related disciplines prefer to work in upper-level hospitals in cities rather than in health centers in rural locations because they have more part-time job opportunities there to complement the low salaries from government employment [6, 10].

Interventions providing extra incentives in rural areas are another possible strategy to encourage non-local health workers to practice in rural regions. Furthermore, the government might also consider the adoption of clinical rotations in rural areas while the students are still in school. Revision of curricula that reflect rural health issues would help students to develop the confidence to practice in rural locations and to be familiar with the medical problems in rural areas. For current rural health workers, concerns about maintenance of knowledge and skills should also be considered because such skills might regress due to shortage of supervision and relative isolated practicing environment. Therefore, continuing education programs should be developed for health personnel in rural regions. It is likely more effective to implement a well-designed integration of interventions including both short-term and long-term ones rather than introduce interventions in an uncoordinated and ad hoc fashion.

#### Improving the health management capacity at the sub-national levels

The inadequate health management capacity especially at the sub-national levels explored in this study is also important since implementation of health workforce interventions relies strongly on competent human resource management [20]. One possible reason for weak health management capacity at the sub-national levels in the Lao People’s Democratic Republic could be that the majority of provincial and district health managers are medical doctors without appropriate management training [10]. Another important reason reported was the decentralization of the health system had happened faster than local management capacity building. Related to this, there is also a lack of support from the central level, i.e., there were no guidelines provided on how to plan and budget at the sub-national level for provincial and district managers for many years after the health system was decentralized [6]. Therefore, resources need to be invested for improving human resource management capacity at all levels. In rural areas, supervision capacity for creating a supportive working environment to retain health professionals needs to be improved through management development initiatives.

#### Strengthening the evaluation of interventions implemented

The Laotian government has implemented certain interventions to address the problems in the national health workforce. The government issued the HRH policy in 2006 to provide incentives for attracting and retaining health workers in remote areas with specific reference to the 47 poorest districts [25]. Moreover, the training of health workers was decentralized to the provinces in order to promote the recruitment and retention of staff closer to their homes in 2009 [6]. In 2012, the government launched a new policy which required medical graduates in medicine, nursing, midwifery, pharmacy, and dentistry to compulsorily serve for 3 years in rural areas before they get licenses to practice [26]. This policy also provides incentives to well-performing health staff in rural areas and attracts new graduates to continue their service providing in the rural area after the required service [26]. The government plans to gradually increase the number of civil servant positions in health facilities, especially for rural sites, and provide civil servant positions to the current contracted health workers; the number of new positions would be 4000 in 2013. However, there is so far no monitoring or evaluation plan in place. Interventions may have different impacts when they are implemented at different sites, and the implementation of experiences from other countries into the Lao People’s Democratic Republic may lead to unexpected outcomes. Appropriate monitoring and evaluation should be considered an essential aspect because the experiences and lessons generated from the intervention implemented in the Lao People’s Democratic Republic could be very valuable.

### Strengths and limitations

This is the first study using qualitative methodology to explore and better understand the problems related to the health workforce in the Lao People’s Democratic Republic. A range of different key stakeholders with insights into the health workforce issues in the Lao People’s Democratic Republic was included, and their rich perspectives were captured. Ten topics extracted from the working life-span framework were adapted into topic guides and probed to gather comprehensive information on the health workforce.

Several limitations should be mentioned. Local key informants from three districts of two provinces were recruited in this research because of the financial and time constraints, some information on the local circumstances may not represent the whole country, but sufficient qualitative information on the national situation of the health workforce was collected from other key informants. Additionally, an interpreter was employed during the in-depth interviews, and it was sometimes difficult for the interpreter to summarize all participant responses. Most IDIs were audio recorded so that the main responses were still captured. In addition, questions on monitoring and evaluation from the topic guides created very little discussion. It may be more appropriate to use quantitative methods to collect evaluation-type data or analyze existing data.

## Conclusions

This study has identified the problems and challenges for the health workforce in the Lao People’s Democratic Republic and explored underlying causes and interrelationships among the problems. It is necessary to understand this situation and the key challenges relating to the health workforce in the Lao People’s Democratic Republic before implementing new policies and interventions.

To improve the distribution of health workers in rural areas, strategies of increasing production and strengthening retention should be well integrated for better effectiveness. Meanwhile, it is essential to take Laotian-specific context into consideration during intervention development and implementation. Furthermore, the government should acknowledge the inadequate health management capacity and invest resources to improve human resource management capacity at all levels. In rural areas, the supervision capacity for creating a supportive working environment to retain health professionals also needs to be improved through management development initiatives. Also, assessment of interventions of health workforce strengthening should be developed as early as possible to synthesize the experiences and lessons in the Lao People’s Democratic Republic.

Due to the complexity of this issue, one study is not sufficient. More quantitative and qualitative studies are required to better and more comprehensively describe and understand the health workforce situation in the Lao People’s Democratic Republic. As the Lao People’s Democratic Republic strives to improve its health workforce, research should also focus on and evaluate the effectiveness of implemented interventions.

## Additional files

Additional file 1: The structure of healthcare facilities in the Lao People’s Democratic Republic, 2012. The number of healthcare facilities at different levels in the Lao People’s Democratic Republic in 2012. (DOCX 12 kb)
Additional file 2: The main points for probing in topic guides. The main points included in the topic guides to cover the key aspects in the working life-span framework. (DOCX 15 kb)

## Abbreviations

DHOs: District health offices
IDI: In-depth interview
MOH: Ministry of Health
NIOPH: National Institute of Public Health (Lao People’s Democratic Republic)
PHC: Primary healthcare
PHOs: Provincial health offices
WHO: World Health Organization
