# Supplementary material for: Challenges for strengthening the health workforce in the Lao People’s Democratic Republic: perspectives from key stakeholders
Source: Hum Resour Health. 2016 Nov 29;14:72. doi: 10.1186/s12960-016-0167-y (PMC5129208; doi:10.1186/s12960-016-0167-y)
Supplement: Additional file 1: — The structure of healthcare facilities in the Lao People’s Democratic Republic, 2012. The number of healthcare facilities at different levels in the Lao People’s Democratic Republic in 2012. (DOCX 12 kb) [file 12960_2016_167_MOESM1_ESM.docx]

**Appendix 1: The structure of healthcare facilities in Laos, 2012**

| **Type of healthcare facilities** | **Number** |
| --- | --- |
| **Public sector** | |
| Special treatment centers (providing dermatology, ophthalmology, and rehabilitation) | 3 |
| Central hospitals | 4 |
| Regional hospitals | 4 |
| Provincial hospitals | 12 |
| District hospitals | 130 |
| Health centers | 894 |
| Village drug kits | 5000 |
| **Private sector** | |
| Hospital | 0 |
| Clinics | 222 |
| Pharmacies | 1993 |

*Data source:[4]*
