# Supplementary material for: Challenges for strengthening the health workforce in the Lao People’s Democratic Republic: perspectives from key stakeholders
Source: Hum Resour Health. 2016 Nov 29;14:72. doi: 10.1186/s12960-016-0167-y (PMC5129208; doi:10.1186/s12960-016-0167-y)
Supplement: Additional file 2: — The main points for probing in topic guides. The main points included in the topic guides to cover the key aspects in the working life-span framework. (DOCX 15 kb) [file 12960_2016_167_MOESM2_ESM.docx]

**Appendix 2: The main points for probing in topic guides**

| Components from framework | | Main probing points in topic guides | | | | | | | | |
| --- | --- | --- | --- | --- | --- | --- | --- | --- | --- | --- |
|  |  | Current stock and structure | Planning and demand | Personal incentives | Performance and performance appraisal | Education + continuing education | Recruitment and hiring | Attraction, retention, and loss | Financial support | Monitoring and evaluation |
| Entry: Preparing the workforce | Planning |  |  |  |  |  |  |  |  |  |
|  | Education |  |  |  |  |  |  |  |  |  |
|  | Recruitment |  |  |  |  |  |  |  |  |  |
| Workforce: Enhancing worker performance | Supervision |  |  |  |  |  |  |  |  |  |
|  | Compensation |  |  |  |  |  |  |  |  |  |
|  | System supports |  |  |  |  |  |  |  |  |  |
|  | Lifelong learning |  |  |  |  |  |  |  |  |  |
|  | Performance |  |  |  |  |  |  |  |  |  |
| Exit: Managing attrition | Migration |  |  |  |  |  |  |  |  |  |
|  | Career choice |  |  |  |  |  |  |  |  |  |
|  | Health and safety |  |  |  |  |  |  |  |  |  |
|  | Retirement |  |  |  |  |  |  |  |  |  |

Note: The cells with green color represent the main areas covered in the topic guides.
